# External application of liver compresses to reduce fatigue in patients with metastatic cancer undergoing radiation therapy, a randomized clinical trial

**DOI:** 10.1186/s13014-021-01757-x

**Published:** 2021-04-19

**Authors:** Pirus Ghadjar, Wiebke Stritter, Irina von Mackensen, Felix Mehrhof, Clara Foucré, Vincent H. Ehrhardt, Marcus Beck, Pimrapat Gebert, Goda Kalinauskaite, Jacqueline S. Luchte, Carmen Stromberger, Volker Budach, Angelika Eggert, Georg Seifert

**Affiliations:** 1 Department of Radiation Oncology, Charité Universitätsmedizin Berlin, corporate member of Freie Universität Berlin, Humboldt-Universität zu Berlin and Berlin Institute of Health, Augustenburger Platz 1, 13353 Berlin, Germany; 2 Division of Oncology and Hematology, Department of Pediatrics, Charité Universitätsmedizin Berlin, corporate member of Freie Universität Berlin, Humboldt-Universität zu Berlin and Berlin Institute of Health, Berlin, Germany; 3 Institute of Biometry and Clinical Epidemiology, Charité Universitätsmedizin Berlin, corporate member of Freie Universität Berlin, Humboldt-Universität zu Berlin and Berlin Institute of Health, 13353 Berlin, Germany; 4 Faculty of Medicine, Department of Paediatrics, University of São Paulo, São Paulo, Brazil

**Keywords:** External application, Compress, Cancer, Metastasis, Radiation therapy, Fatigue, Integrative medicine

## Abstract

**Background:**

Liver compresses are frequently used in integrative medicine as supportive therapy during cancer treatment in order to reduce fatigue. We performed a pilot study to test whether the external application of yarrow liver compresses impacts fatigue in patients with metastatic cancer undergoing radiation therapy.

**Methods:**

A randomized prospective pilot trial was performed including patients with brain metastasis or bone metastasis of solid tumors. Patients underwent either palliative radiation therapy (RT) of the metastatic lesions (control group) over two weeks or the same RT with additional external application of yarrow liver compresses once daily during RT. The primary objective was improvement on the general fatigue subscale of the multidimensional fatigue inventory (MFI-20) at the end of treatment, where a mean difference of two points is considered clinically relevant. Secondary objectives included psychological distress, quality of life and qualitative analysis with self-established visual analogue scales (VAS). Mean differences in general fatigue at the end of treatment compared to baseline were analyzed using the ANCOVA test.

**Results:**

From 09/2017 to 08/2019 a total of 39 patients were randomized. Due to drop outs 24 patients (12 per group) were available for analysis. Patients in the intervention group received a mean number of 10.5 (range, 7–14) applications of yarrow liver compresses. The mean improvement at the end of therapy on the general fatigue subscale of the MFI-20 was 2 points in favor of the intervention group (*p* = 0.13), and all other MFI-20 subscales showed at least a trend towards improvement in favor of the intervention group. Likewise, psychological distress and VAS data was improved, the latter reaching statistical significance for the symptoms fatigue, tension and lack of drive. Major toxicities were not observed.

**Conclusions:**

External application of liver compresses appears to reduce fatigue within a clinical relevant range in patients with metastatic cancer undergoing radiation therapy.

*Trial registration*: ISRCTN, ICTRP DRKS00012999

**Supplementary Information:**

The online version contains supplementary material available at 10.1186/s13014-021-01757-x.

## Background

Fatigue has been described to be present in ≥ 30% of cancer patients and adversely impacts upon quality of life (QoL) [1]. The causation of fatigue is complex and often multifactorial, and treatment exists only for those patients in whom the cause can be targeted therapeutically, e.g. in patients with anemia causing fatigue who can receive transfusions. When no causal treatment for fatigue is available, across-the-board approaches such as increase of physical activity, treatment of possible sleep disorders and adherence to a balanced diet are advised, commonly only with modest success.

Randomized trials have demonstrated fatigue reduction through various interventions such as yoga [2], eurythmy therapy [3] and sport [4], but these interventions are not regularly performed.

Patients who undergo palliative radiation therapy (RT) for brain or bone metastases are commonly frail patients suffering symptoms caused by the disease and the treatment and fatigue has been shown to increase during and three months after RT [5, 6].

Yarrow (Achillea millefolium) is a medical plant which has been described as an analeptic and is perceived as warming, anticonvulsant and tonic according to largely unconfirmed anthroposophic medicine theories originating from Germany [7–9]. However, clinical trials are lacking on this topic [10]. Nevertheless, yarrow liver compresses are commonly used in integrative medicine as supportive therapy during cancer treatment due to its hepatoprotective qualities, amongst others to reduce fatigue. While aspects of palliative care already strongly impact on daily practice in radiation oncology [11] skepticism exists towards integrative approaches and its effects [12].

We conducted a prospective pilot trial to explore the potential fatigue improving effect of an external application of yarrow liver compresses in metastatic cancer patients undergoing RT.

## Patients and methods

### Patient selection

This study was approved by the Ethical Committee of the Charité Universitätsmedizin Berlin, Germany (Reference number EA1/078/17) and the pilot trial was registered in the Cochrane Central Register of Controlled Trials (DRKS00012999).

For logistical reasons the decision was made to conduct this trial among patients with metastatic cancer undergoing radiation therapy being on an inpatient basis within the Department of Radiation Oncology, Charité Universitätsmedizin Berlin.

The criteria for patient eligibility were age ≥ 18 years, with at least minor fatigue according to the general fatigue subscale of the multidimensional fatigue inventory (MFI-20) [13], estimated life expectancy > 3 months, Karnofsky performance score ≥ 60%, indication for inpatient palliative whole brain radiation therapy (WBRT) or palliative analgesic RT of bone metastasis. Patients were required to be inpatients due to the foreseen logistic hurdles in preparing and applying liver compresses in an outpatient setting. Patient written consent was mandatory. Patients were ineligible when severe psychiatric disorders or allergies prohibited participation and in cases with medical conditions potentially causing fatigue such as severe hypothyroidism, sleep apnea, insomnia, anemia with hemoglobin levels < 8 g/dL, cachexia with a body mass index < 18.5, chronic kidney failure or acute depression. Patients with liver metastasis were also ineligible because in this case warming compresses were contraindicated, as were patients participating in other clinical trials. Patients receiving both WBRT and bone RT were also ineligible.

### Treatment

Patients in both groups were treated by external beam RT. All patients underwent a dedicated planning computed tomography scan. For WBRT patients received 10 fractions of 3 Gy over 2 weeks using a three-dimensional conformal RT (3D-CRT) approach, wearing a thermoplastic mask for immobilization. Patients with bone metastasis received either 10 fractions of 3 Gy over 2 weeks or a slightly intensified regimen in the case of soft tissue tumor mass with up to 12–13 fractions of 3 Gy using 3D-CRT or an intensity modulated radiation therapy (IMRT) or volumetric modulated arc treatment (VMAT) approach. For WBRT the brain was defined as gross tumor volume (GTV) surrounded by a 5 mm margin to establish the planning target volume (PTV). For the treatment of bone metastasis, the involved bones/bone regions were defined as GTV and a 5–10 mm margin added to establish the PTV. RT was performed using a 6-MV linear accelerator with multileaf collimators (Varian, USA).

During the inpatient course of treatment patients in both groups were offered palliative care in the form of visits from a dedicated multi-professional palliative care team of physicians, nurses, psycho-oncologists, physiotherapists and social workers, depending on the requirements of the respective patient.

**Fig. 1:**
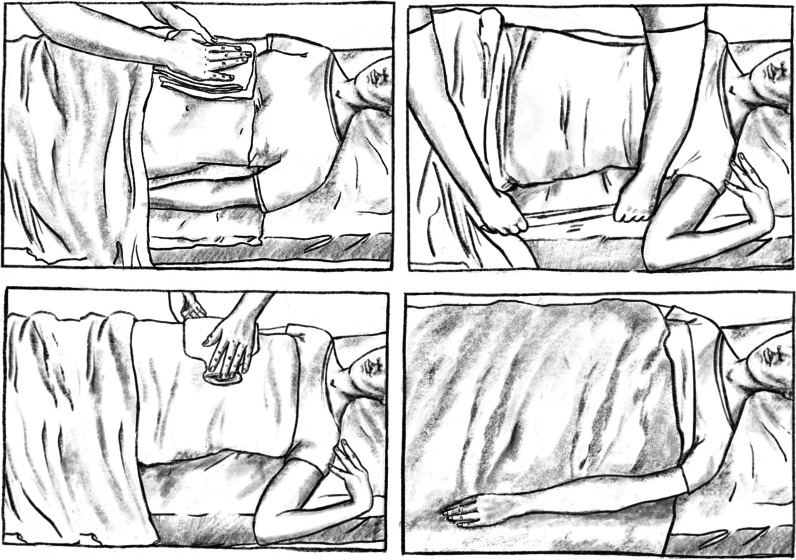
Step-wise external application of a yarrow liver compress with careful placement of the inner muslin cloth, which had been doused with a 60 °C hot yarrow infusion and then firmly wrung out, and the cotton compress cloth (on top of the inner cloth) on the right upper abdomen and lower costal arch of the comfortably positioned patient (top left); the inner and compress cloth were then covered with an outer wrap underneath the patient’s back which was folded over from both sides (top right); a hot-water bottle was placed on top of the compress (bottom left); the patient was then covered with a blanket and allowed to rest for a total duration of 50–55 min (bottom right)

External application of yarrow liver compresses was performed once daily over the course of two weeks of RT, the minimum number of applications was set to seven. Applications were preferably performed after lunch or in the evening. The liver compresses were prepared and applied by trained members of our scientific team in cooperation with the staff on the ward. For preparation of the compresses a dedicated room on the in-patient ward outside the patient room was used. One tablespoon of yarrow tea with cut blossoms, stems and leaves of common yarrow was infused with 500 ml boiling water and brewed for 10 min. The infusion was then strained and cooled for 5 min at room temperature to reach the required application temperature of 60 °C. A muslin cloth (made of cotton) was folded to the size of 20 × 30 cm, rolled up firmly (into a small roll) and placed on a rough cotton cloth (such as a tea towel). Both together were rolled up tightly, twisting the rough cotton cloth so that the two ends formed handles. This roll was then placed in a bowl. The necessary materials where brought to the patient’s room where the patient was then comfortably positioned on the bed lying on an outer wrap. The wound-up roll in the bowl was then doused with the 60 °C hot yarrow infusion and wrung out firmly, wearing gloves to protect the hands from the heat. The muslin cloth was removed, carefully placed on the patient’s right upper abdomen and lower costal arch. In case the patient experienced the muslin cotton as too hot, it was fanned to make the heat bearable. It was then covered with a cotton compress cloth which had been previously folded to the size of 20 × 30 cm. The outer wrap underneath the patient’s back was then folded tightly over the compress from both sides. A hot-water bottle was placed on top and could be removed at the patient’s convenience. The patient was then covered with a blanket and allowed to rest for 20–25 min. Care was taken to leave the patient alone in the room without external disturbance during the rest period. After 20–25 min the moist inner muslin cloth was removed while the compress cloth and outer wrap stayed in place, including the hot-water bottle and the patient left to rest for another 30 min (Fig. 1).

### Measurements and endpoints

Fatigue was assessed at baseline, after one week and at the end of the treatment according to the German version of the MFI-20 [13]. The MFI-20 is a 20-item psychometric inventory designed to evaluate five dimensions of fatigue: general fatigue, physical fatigue, reduced motivation, reduced activity, and mental fatigue [14]. The primary trial endpoint was the general fatigue subscale of the MFI-20 at the end of treatment. A mean difference of two points compared to baseline was considered clinically relevant [15].

Secondary endpoints included psychological distress as measured according to the German version of the distress thermometer of the National Comprehensive Cancer Network (NCCN) [16], patient’s quality of life was assessed according to the European Organization for Research and Treatment of Cancer (EORTC) QLQ-C30 questionnaire [17]. Moreover, a qualitative analysis of patients of the intervention group was performed using questions regarding their condition on self-established visual analogue scales (VAS). The VAS covered tension, restlessness, fatigue, lack of drive, exhaustion, experience of warmth (cold hands/feet) and pain. All questionnaires were administered at baseline, after one week of treatment and at the end of treatment. As a sub-project, physiological data was assessed using heart rate variability and body temperature, which will be reported separately. Liver compress-related symptoms were assessed after each application and the time of onset and resolution as well as the intensity (mild vs. intermediate vs. severe) was noted.

### Statistical analyses

The trial was designed as a randomized, non-blinded prospective clinical pilot trial. A pilot phase was planned with subsequent sample size recalculation. Block randomization was performed with stratification according to the RT site brain vs. bone.

The power calculation for the pilot phase was based on a 3-point reduction on the general fatigue subscale of the MFI-20 in the intervention group vs. control group at the end of treatment compared to baseline. With a common standard deviation of 3 points, alpha 0.05 and a power of 80% an initial sample size of 34 patients (17 per group) was required for an unpaired t-test. Accounting for 10% drop-outs 38 patients should initially be included. Due to the unblinded non-confirmatory design were p-values are regarded as exploratory, no alpha-adjustment was planned. The sample size calculation was performed using G*Power Version 3.1.3.

Analysis of the pilot trial was performed using analysis of covariance (ANCOVA) with end and baseline differences on the general fatigue subscale as dependent and group variables, RT site brain vs. bone (strata) as independent variables and baseline general fatigue as covariate variable. A *p* value < 0.05 will be regarded as significant. *P*-values were not corrected for multiple testing. The effect size partial eta squared (*η*^2^) was presented to show the difference between groups as well as omega squared (*ω*^2^) for correcting biased from a small sample size [18].

After the pilot trial had experienced severe accrual problems, due to many patients being ineligible and several other patients withdrawing from the trial after inclusion (Fig. 1), the pilot trial was halted in September 2019. Given the mean difference of two points on the general fatigue subscale (considered clinically relevant) in favor of the intervention group already found, the trial team decided to stop the pilot trial and moved ahead to analyze the results. All statistical tests were performed using Stata IC15 (StataCorp, 2017, College Station, TX, USA).

### Results

**Fig. 2:**
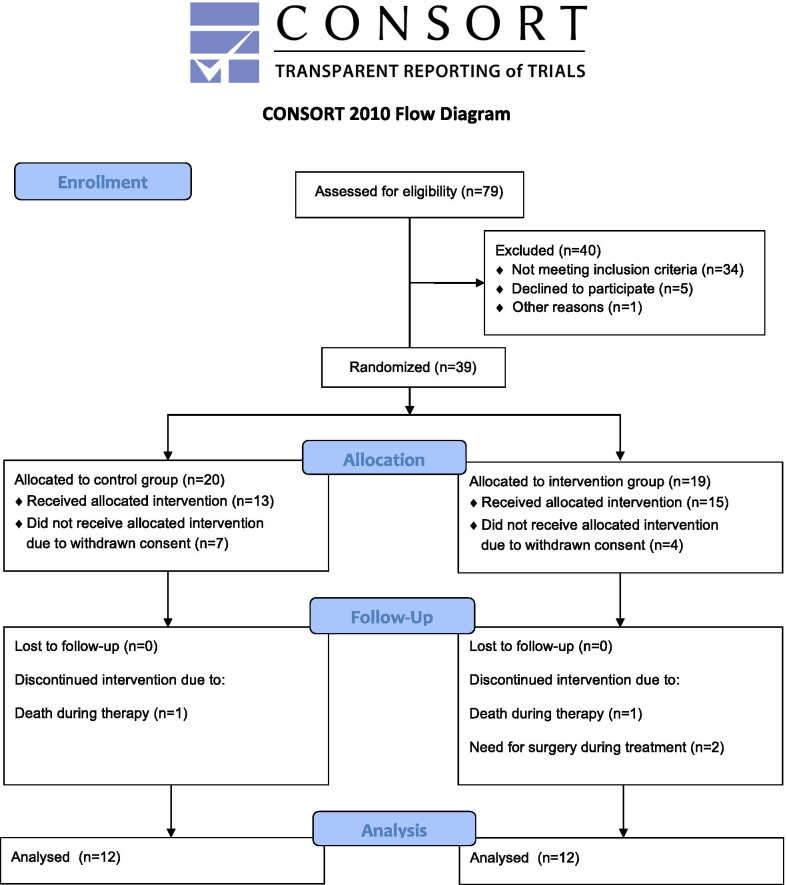
Consort diagram of the trial

Between September 2017 and August 2019 a total of 79 preselected patients were screened, of whom 39 were randomized, and after exclusion of drop outs a total of 24 patients (12 per group) were available for analysis (Fig. 2). The baseline patient characteristics are summarized in Table 1, there were no significant differences between the two pilot trial groups. Patients in the intervention group received a mean number of 10.5 applications of yarrow liver compresses (range 7–14).

**Table 1:** Patient characteristics

	Radiotherapy alone (n = 12)	Radiotherapy and Yarrow compress (n = 12)	Total [24]
Gender			
Male	3 (25%)	3 (25%)	6 (25%)
Female	9 (50%)	9 (50%)	18 (75%)
Age (years)			
Median (min.–max.)	60.5 (49–83)	56.5 (35–68)	58.5 (34–83)
Karnofsky-Index			
80–100	4 (33%)	3 (25%)	7 (30%)
70	5 (42%)	4 (33%)	9 (37%)
60	3 (25%)	5 (42%)	8 (33%)
Site of RT			
Bone	6 (50%)	7 (58%)	13 (54%)
Brain	6 (50%)	5 (42%)	11 (46%)
Baseline Fatigue*			
Median (min.-max.)	14 (10–20)	16 (10–20)	14.5 (10–20)

^*^Based on the general fatigue subscale of the multidimensional fatigue inventory (MFI-20)

**Fig. 3:**
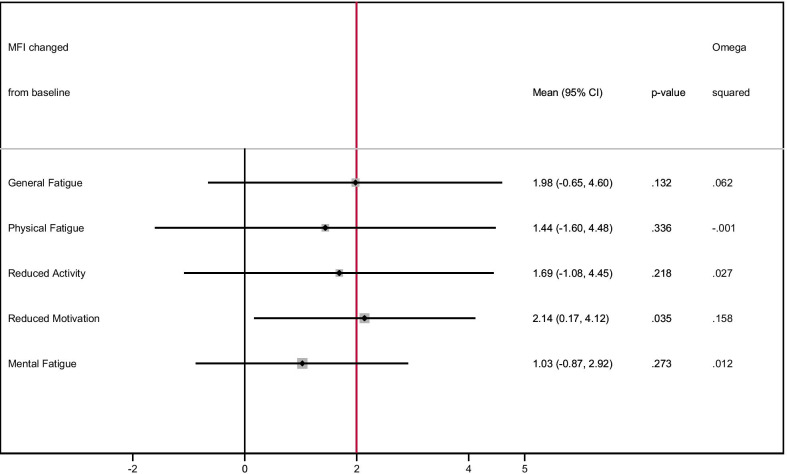
Plot showing the mean difference between groups of the change from baseline to end of treatment for each subscale of the MFI-20 after adjustments for baseline values and 95%CI. Red line shows the clinically relevant difference of two points

For the primary endpoint, the general fatigue subscale of the MFI-20, we observed a mean improvement over baseline at the end of treatment of 2 points in favor of the intervention group (*p* = 0.13, effect size (ω^2^) = 0.062) (Table 2 and Fig. 3). All other MFI-20 subscales tended in the same direction towards improvement of fatigue in patients of the intervention group. For the MFI-20 subscale reduced motivation the mean improvement at the end of therapy was 2.1 points the difference being statistically significant between the pilot trial groups (p = 0.035, effect size (ω^2^) = 0.158) (Fig. 3). Mean changes from baseline (t0) at one week (t1) and at the end of treatment (t2) for all MFI-20 subscales are provided in the Additional file 1: Fig. 4.

**Table 2:** Comparison of fatigue and other patient reported outcomes between the trial arms

	Intervention	Control	Mean difference* (I–C)	95% CI	p-value	Effect size (Partial η^2^)	Effect size (ω^2^)	
	Mean (SD)	Mean (SD)		Lower	Upper			
**MFI subscales (T0–T2)**	(n = 12)	(n = 12)						
General Fatigue	1.08 (2.57)	−0.50 (3.75)	1.98	−0.65	4.60	0.132	0.110	0.062
Physical Fatigue	0.83 (4.17)	0.42 (4.74)	1.44	−1.60	4.48	0.336	0.046	−0.001
Reduced Activity	1.75 (2.96)	0.58 (4.68)	1.69	−1.08	4.45	0.218	0.075	0.027
Reduced Motivation	0.08 (3.06)	−2.58 (2.43)	2.14	0.17	4.12	0.035	0.204	0.158
Mental Fatigue	1.25 (3.31)	0.00 (4.09)	1.03	−0.87	2.92	0.273	0.060	0.012
**Psychological Distress (T0–T2)**	(n = 12)	(n = 12)						
Distress	0.82 (2.27)	−0.15 (2.24)	1.08	−0.70	2.87	0.218	0.088	0.032

**VAS (T2—T0)**	(n = 11)	(n = 10)						
VAS1 Tension	0.64 (1.29)	−0.80 (1.42)	1.14	0.04	2.25	0.044	0.219	0.165
VAS2 Restlessness	0.55 (1.04)	−0.35 (1.13)	0.77	−0.10	1.64	0.080	0.169	0.115
VAS3 Fatigue	0.64 (1.38)	−0.60 (0.81)	1.12	0.25	1.98	0.015	0.301	0.250
VAS4 Lack of drive	0.36 (1.63)	−0.45 (1.26)	0.97	0.12	1.82	0.028	0.253	0.201
VAS5 Exhaustion	0.41 (1.18)	−0.20 (0.79)	0.76	−0.05	1.57	0.063	0.189	0.135
VAS6 Cold hands/feet	0.32 (1.71)	0.15 (0.91)	0.25	−0.77	1.28	0.608	0.016	−0.040
VAS7 Pain	0.95 (1.67)	−0.10 (1.51)	0.75	−0.48	1.99	0.215	0.089	0.034

**EORTC QLQ-C30 (T2–T0)**	(n = 12)	(n = 12)						
Global health status/QoL	6.94 (25.08)	5.56 (12.97)	2.66	−11.71	17.02	0.703	0.007	−0.040

MFI = multidimensional fatigue inventory; SD = standard deviation; I-C = Intervention group—Control group; CI = confidence interval; η^2^ = Partial eta squared; ω^2^ = Omega squared; VAS = visual analogue scale; EORTC = European Organization for Research and Treatment of Cancer; QLQ = quality of life questionnaire; QoL = quality of life; * Mean difference and 95%CI were calculated using ANCOVA with adjustment for baseline and RT sites (brain vs. bone)

Differences in psychological distress also tended to favour the intervention group (Table 2). Moreover, the VAS results showing significant improvement in the symptoms fatigue, tension and lack of drive in patients from the intervention group when compared to the control group also suggested a positive effect of the intervention (Table 2). There was no difference in the global health status/quality of life between the two groups (Table 2).

There were no significant differences between patients treated with WBRT or bone metastasis with respect to reduction of fatigue.

In five patients from the intervention group (42%) local redness of the skin was observed at least once after the application of the compress which was not perceived as uncomfortable by the patient and completely returned to normal without intervention. One patient developed mild nausea and in another patient a single episode of temporary localized crampy pain in the liver region was observed after application of the compress. Treatments were continued in both patients and neither of the two symptoms occurred again. Major compress-related toxicities were not observed.

## Discussion

In this pilot trial we observed a clinically relevant reduction of fatigue after external application of yarrow liver compresses in patients with metastatic cancer undergoing RT. This is significant given the small cohorts available for analysis. All subscales of the MFI-20 tended in the same direction and other measures such as the psychological distress and VAS data were also supportive of the assumption that there is a true effect. In addition, it appears that yarrow liver compresses were tolerated well by patients with no major toxicity observed.

Patients undergoing WBRT for brain metastasis have a life expectancy of only 3–6 months and commonly experience intracranial recurrence and or recurrence of the extracranial disease [19]. Likewise, pain progression at the treated site and/or development of new bone metastasis are relatively common in patients with bone metastasis [20, 21]. As these RT treatments, subsequent salvage RT treatments as well as additional systemic treatments can cause fatigue, and as patient reported symptoms at baseline were shown to predict survival [22], the identification of fatigue reducing well tolerated and cost effective strategies is very important. The time required for preparation of a yarrow liver compress is estimated to be 5–10 min without significant material expenses. In this respect, it is a simple method that can be applied quickly, and which certainly has a calming effect on patients and could thus save costs and time overall. Since other studies point to the anti-inflammatory and spasmolytic effect of yarrow [23–25], these patients might profit from this simple intervention on multiple levels.

Because of problems with patient recruitment the pilot trial was prolonged for a total duration of 2 years but its continuation was subsequently impossible. On account of the pilot nature of this trial the decision was made to perform early data analysis. A high number of screened patients could not be included in the pilot trial as they did not meet the inclusion criteria of at least minor fatigue. This needs to be accounted for in a subsequent trial in another group of cancer patients. However, it is unknown whether yarrow liver compresses might even be more effective in a patient population with a higher degree of baseline fatigue.

It is also not known, relating to the non-blinded character of our pilot trial, whether the observed fatigue reduction was caused by the yarrow itself, or the application of the liver compresses, due to the attention towards the patient during the whole procedure, due to a placebo effect or due to a combination of the above. Exploring the exact mechanism of action is somewhat unimportant from a patient perspective as patients do appear to benefit from the compress. However, from a scientific point of view it might be worth performing a placebo-controlled trial e.g. liver compress with and without yarrow, to further expand the knowledge on external applications.

## Conclusions

External application of liver compresses appears to reduce fatigue in patients with metastatic cancer undergoing palliative radiation therapy.

## Supplementary Information

**Additional file 1.** **Figure 4**: Line plot showing mean changes from baseline (t0) at one week (t1) and at end of treatment (t2) for all MFI-20 subscales after adjustments for different baseline values.

## Data Availability

The datasets used and/or analyzed during the current study are available from the corresponding author on reasonable request.

## Abbreviations

CI: Confidence interval
EORTC: European Organization for Research and Treatment of Cancer
GTV: Gross tumor volume
I-C: Intervention group – Control group
IMRT: Intensity modulated radiation therapy
MFI: Multidimensional fatigue inventory
NCCN: National Comprehensive Cancer Network
PTV: Planning target volume
RT: Radiation therapy
SD: Standard deviation
VAS: Visual analogue scale
VMAT: Volumetric modulated arc treatment
WBRT: Whole brain radiation therapy
QLQ: Quality of life questionnaire
QoL: Quality of life
3D-CRT: Three-dimensional conformal RT
