# Supplementary figures and images for: External application of liver compresses to reduce fatigue in patients with metastatic cancer undergoing radiation therapy, a randomized clinical trial
Source: Radiat Oncol. 2021 Apr 19;16:76. doi: 10.1186/s13014-021-01757-x (PMC8054395; doi:10.1186/s13014-021-01757-x)

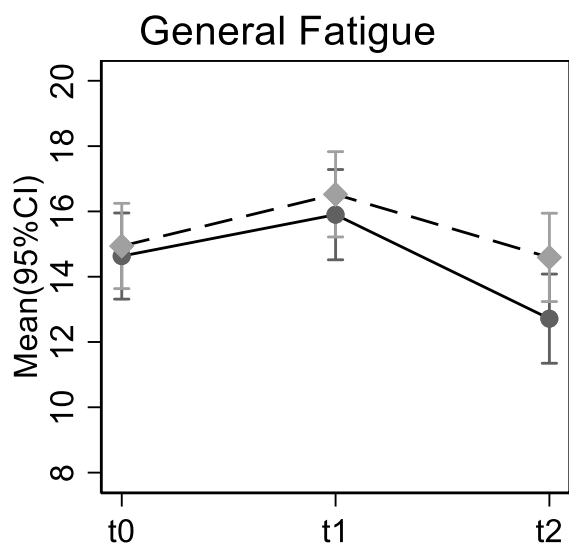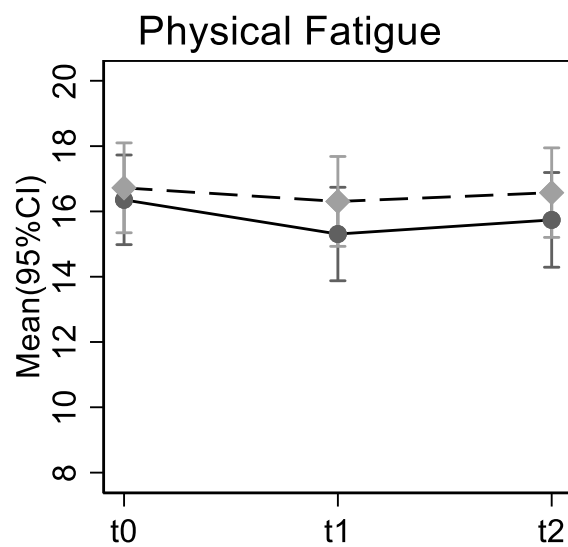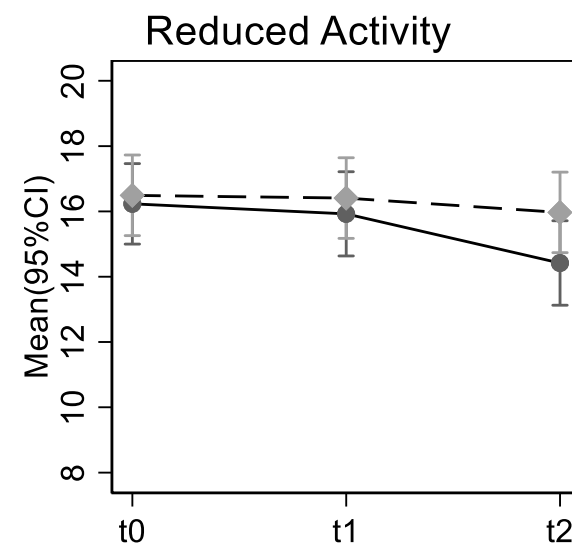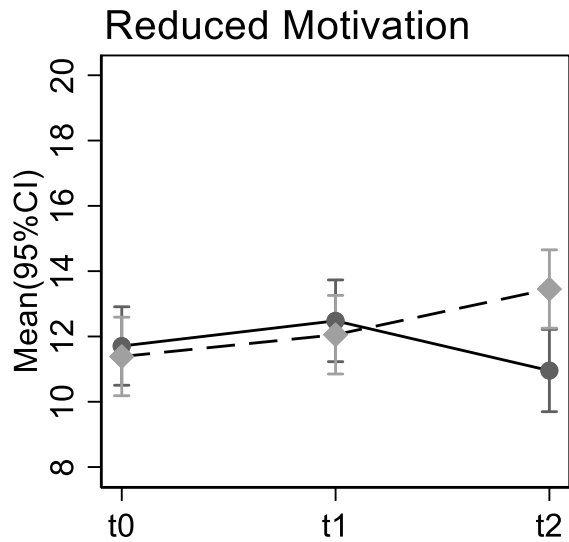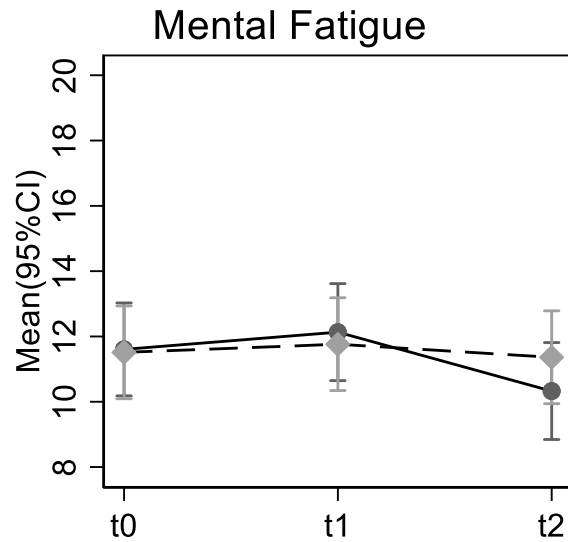

—●— Intervention group  
- -◇- - Control group

Supplement: Supplementary file 1 — Additional file 1. Figure 4: Line plot showing mean changes from baseline (t0) at one week (t1) and at end of treatment (t2) for all MFI-20 subscales after adjustments for different baseline values. [file 13014_2021_1757_MOESM1_ESM.pdf]
